# A new species of *Amentotaxus* (Taxaceae) from China, Vietnam, and Laos

**DOI:** 10.3897/phytokeys.130.33956

**Published:** 2019-08-29

**Authors:** Lian-Ming Gao, Shao-Lin Tan, Gui-Liang Zhang, Philip Thomas

**Affiliations:** 1 CAS Key Laboratory for Plant Diversity and Biogeography of East Asia, Kunming Institute of Botany, Chinese Academy of Sciences, Kunming 650201, China Kunming Institute of Botany, Chinese Academy of Sciences Kunming China; 2 Kunming College of Life Science, University of Chinese Academy of Sciences, Kunming, Yunnan 650201, China University of Chinese Academy of Sciences Kunming China; 3 Hekou Branch of Administration Bureau of Daweishan National Nature Reserve, Hekou, Yunnan 661399, China Hekou Branch of Administration Bureau of Daweishan National Nature Reserve Hekou China; 4 Royal Botanic Garden Edinburgh, 20A Inverleith Row, Edinburgh EH3 5LR, Scotland, UK Royal Botanic Garden Edinburgh Edinburgh United Kingdom

**Keywords:** *Amentotaxus hekouensis*, New species, Endangered species, DNA barcoding, China

## Abstract

A new species *Amentotaxus hekouensis* L.M. Gao is described as new to science from Hekou, Yunnan of China, Lao Cai of Vietnam and Xiang Khouang of Laos. The new species is similar to *A. argotaenia* (Hance) Pilg. in linear or linear-lanceolate leaves, stomatal bands white and microsporophylls 6–8, each with 4–6 pollen sacs, but differs from the latter by its larger leaf size with 8–12.5 cm × 0.9–1.4 cm (vs. 2–11 cm × 0.5–1.1 cm in *A. argotaenia*), long acuminate leaf apex (vs. rounded to sharply triangular in *A. argotaenia*), stomatal bands with 25–30 rows (vs. 15–25 rows in *A. argotaenia*), stomatal bands equal to or slightly narrower than marginal bands (vs. narrower than marginal bands in *A. argotaenia*); pollen-cone racemes borne 1–2 (vs. 2–4 (10) in *A. argotaenia*), cones in 12–16 pairs (vs. ca. 12 pairs in *A. argotaenia*). Its distinctive nature has also been confirmed through DNA barcoding analysis of this genus. The new species is provisionally assessed as endangered (EN) due to its restricted distribution, small population size and the prevalence of habitat destruction within its range.

## Introduction

*Amentotaxus* Pilg. (Taxaceae), comprising of five or six species, is confined to southern China (including Taiwan), small areas of the eastern Himalayas and parts of Indo-China (Farjon 2010). All species of *Amentotaxus* are currently assessed as threatened at either national or global level (Wang and Xie 2004; IUCN 2016a). They usually occur in the understorey of moist submontane and montane semi-deciduous or evergreen forests (Gao et al. 2017). *Amentotaxus argotaenia* Pilg. is the most widespread species in this genus, recorded from most provinces of southern China as well as several areas of Vietnam and Laos (Fu et al. 1999; Farjon 2010; Nguyen et al. 2004; Averyanov et al. 2014). *Amentotaxus yunnanensis* H.L. Li is generally confined to the evergreen forests in the karst formation of southeast Yunnan, southwest Guizhou and adjacent areas of Vietnam and Laos (Fu et al. 1999; Farjon 2010; Averyanov et al. 2014). In 1996, *A. hatuyenensis* T.H. Nguyen was described from a single locality in Hagiang Province, northern Vietnam (Nguyen and Vidal 1996), but it is now regarded as a synonym of *A. yunnanensis*, based on morphological and molecular data (Phan et al. 2014; Gao et al. 2017). The remaining accepted species are restricted to Taiwan of China, eastern Himalayas and central Vietnam.

**Figure 1.**
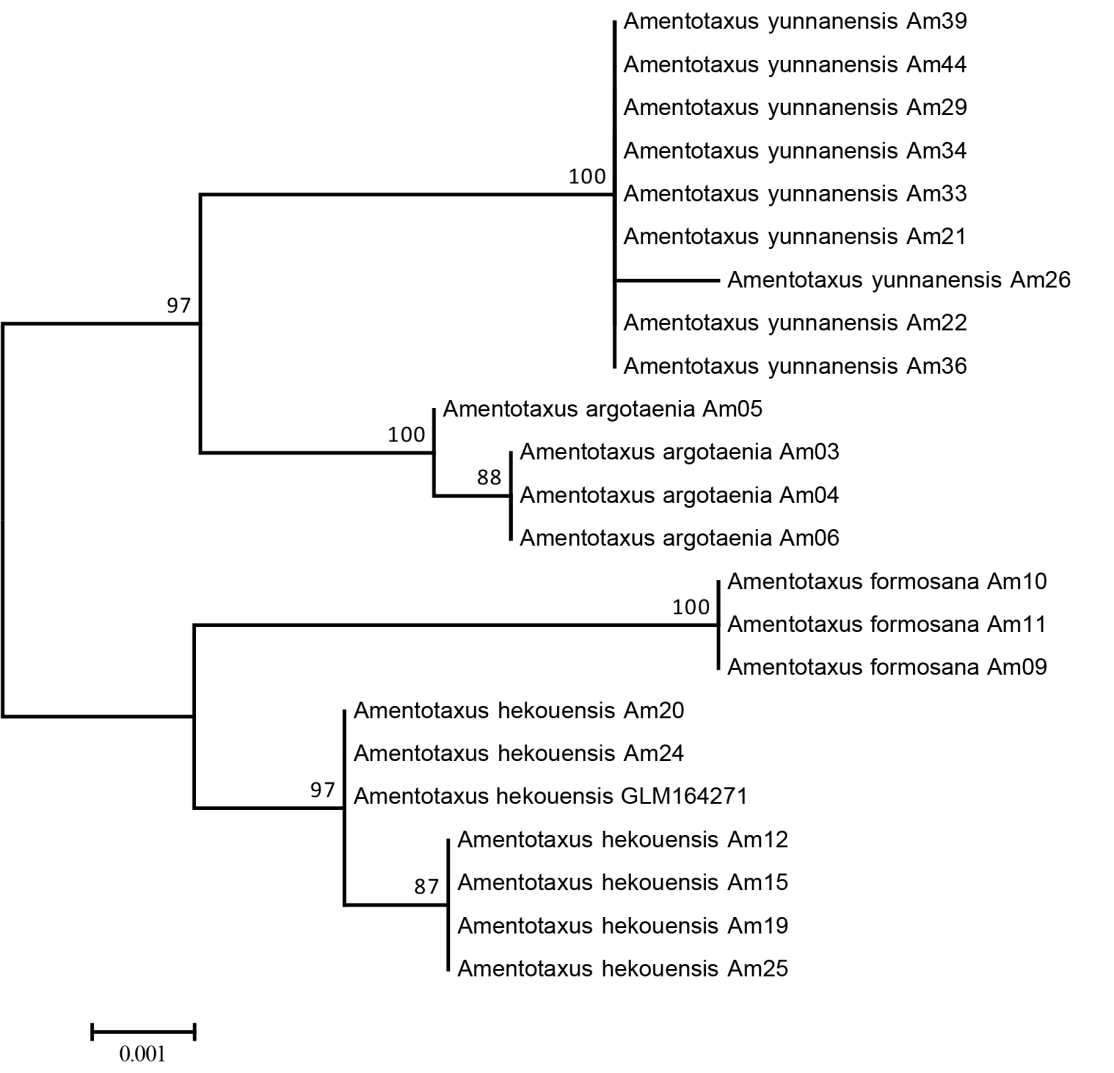
Neighbour-joining (NJ) tree of ITS and *trnL-F* sequences of *Amentotaxus* based on P-distance with bootstrap values above 50%.

In September, 2006, specimens of an *Amentotaxus* tree were collected from limestone montane forest near Longyinchong village, Nanxi town, Hekou county, Yunnan province, China. Its morphology differed markedly from *A. yunnanensis* which commonly occurs in this region. In 2012, a second collection with the same morphology was found in the same region. In our DNA barcoding study (Gao et al. 2017), specimens from Hekou formed a distinct clade with specimens with similar morphology from Lao Cai in Vietnam and Xiang Khoang in Laos. The putative new species differs from all other recognised species in the length and width of the leaves, the shape of the leaf apex, curvature of the leaf margin, stomatal band width and its ratio to the leaf marginal bands. In 2016, several further surveys collected specimens with male cones that showed differences in the number of pollen sacs and the length of the racemes. Data analysis of *trnL-F* and ITS sequences for the newly collected specimen GLM-164271 had an identical sequence with the specimen GLM-06209 (Am 20) and CKF250 (Am 24), used in our DNA barcoding study (Gao et al. 2017), lending further support to the recognition of a new species (Figure 1). Although female cones have not yet been collected, other species in the genus do not show significant differences in the female cones (Fu et al. 1999; Farjon 2010).

## Materials and methods

All measurements of the new *Amentotaxus* species were taken from dried herbarium specimens and field notes. In the description, length and width are represented as length × width. In total, eleven dried specimens of the new species were examined. All measurements of *A. argotaenia* (Hance) Pilg. and *A. yunnanensis* H.L. Li were based on literature (Fu et al. 1999; Farjon 2010) and our observations.

## Taxonomic treatment

### Amentotaxus hekouensis

Taxon classificationPlantaePinalesTaxaceae

L.M. Gao sp. nov.

BF626FA0F2735B619274F4CFD6ADEA0D

urn:lsid:ipni.org:names:60479341-2

**Figure 2.**
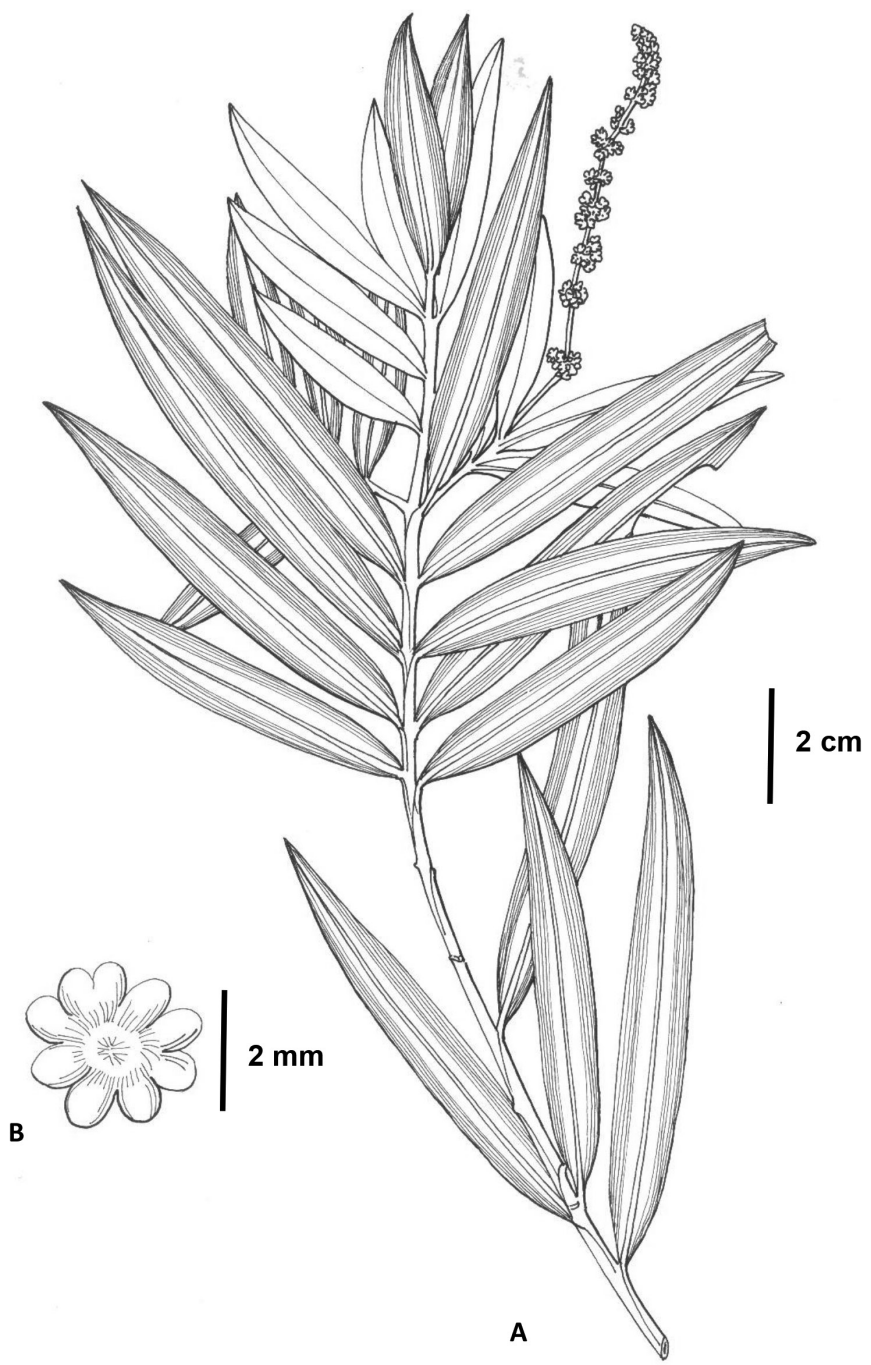
*Amentotaxus hekouensis* L.M. Gao (from the holotype, drawn by Ling Wang). **A** Branchlets with male cone **B** pollen sacs.

**Figure 3.**
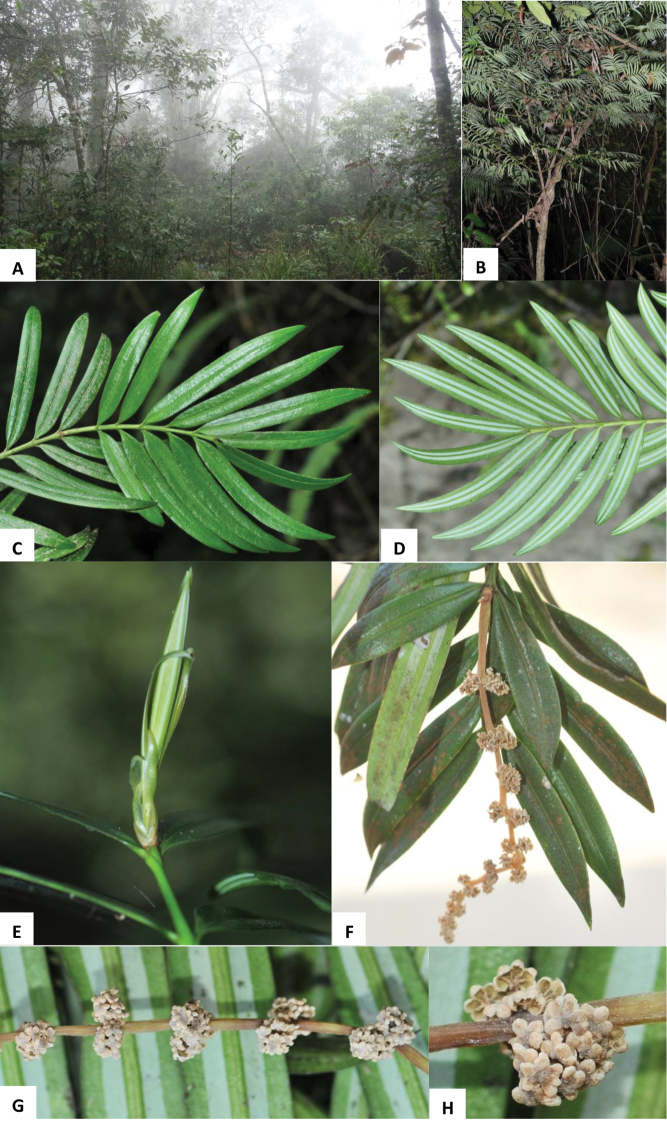
*Amentotaxus hekouensis* L.M. Gao. **A** Habitat **B** habit **C** branchlet with adaxial leaves **D** branchlet with abaxial leaves **E** young shoot **F** branchlet and male cone racemes with microsporophylls **G** male cone racemes **H** microsporophylls and pollen sacs.

Figs 2 , 3 , Table 1

#### Diagnosis.

*Amentotaxus hekouensis* L.M. Gao resembles *A. argotaenia* (Hance) Pilg., but differs in its larger leaf size (8–12.5 cm × 0.9–1.4 cm), long acuminate leaf apex, leaf margin flat with slightly wavy, stomatal bands white or greenish-white with 25–30 rows, stomatal bands equal or slight narrower than marginal bands; pollen-cone racemes borne 1–2, cones in 12–16 pairs, microsporophylls 6–8, each with 4–6 pollen sacs.

#### Type.

CHINA. Yunnan: Honghe, Hekou County, Nanxi Town, Longyinchong village, 22°41'8.9"N, 104°01'11.5"E, 926 m alt., 16 April 2016 (with male cone), *G. L. Zhang, GLM-164271* (holotype: KUN!; isotypes: KUN!)

#### Morphological description.

Small tree to 4–5 m tall; branch cylindric or subtetragonal, grey; leafy branchlets ascending or sub-erect, broadly rectangular to oblong-elliptic in outline, 6–12 × 11–20 cm, axis green in 1^st^ year, greenish-yellow in 2^nd^ and 3^rd^ years, quadrangular or subterete in cross section. Leaves borne at 50–80° to branchlet axis, distichous, twisted at the short petiolate or nearly sessile base, petiole 2–4 mm long, almost opposite, 4–6 leaf pairs on each branchlet; leaves leathery, thin, linear or linear-lanceolate, 8–12.5 × 0.9–1.4 cm, straight, slightly falcate at the apex, cuneate at base, asymmetric, apex long acuminate, leaf margin flat or slightly down-curved, usually slightly wavy; leaf marginal band dark green in fresh, yellowish-green in dry, 2.5–3.2 mm wide; stomatal bands white or greenish-white, 2.1–3.0 mm wide, equal or slight narrower (> 3/4) to marginal bands, 25–30 rows, densely arranged; midvein slight sunken or flat adaxially, raised abaxially, 1.2–2.0 mm wide, narrower than the stomatal bands and marginal bands, green, same colour as the branchlet. Male-cone racemes borne 1–2, ca. 8.0 cm long; cones in 12–16 pairs, ovoid, cones at base maturing earlier than those at apex; microsporophylls 6–8, peltate, each with 4–8 pollen sacs. Ovulate cone and mature seed unknown. Male cones maturing March to April.

#### Additional specimens examined.

CHINA. Yunnan: Hekou County, Nanxi Town, 20 Sept 2006, *L. M. Gao et al. GLM-06209* (KUN!); ibid., 20 Oct 2012, *L.M. Gao GLM-123943* (KUN!), ibid., 31 Jan 2016, *L. M. Gao et al. GLM-164258, GLM-164259* (KUN!), ibid., 21 Oct. 2001, *Y. M. Shui et al. 15078* (KUN); Funing County, Long-may, 24 April 1940, *C.W. Wang 88812* (KUN); Malipo County, 4 Jan. 1940, *C.W. Wang 86175* (KUN). Vietnam. Lao Cai: Muong Khuong, Nam Chay commune, Moi village, 21 Nov. 2009, *CKF team CKF250* (KUN, CPC, CMBG); Hoa Binh: Mai Chau, Pa Co commune, 3 Dec. 2003, *S.G. Wu et al*. *WP413* (KUN). Laos. Xiang Khouang: Phak Leung Mt., Tanh village, Dec. 2008, *X. Gong GX-5* (KUN).

#### Distribution and ecology.

**Figure 4.**
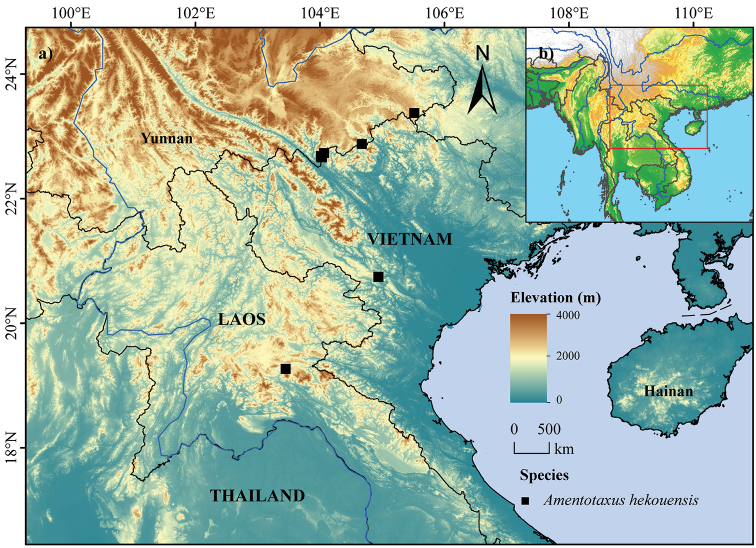
Geographical distribution of *Amentotaxus hekouensis* L. M. Gao.

*Amentotaxus hekouensis* L.M. Gao has only been found in small disjunct areas in China (Funing, Malipo and Hekou County, Yunnan Province), Vietnam (Muong Khuong, Lao Cai Province; Mai Chau, Hoa Binh Province) and Laos (Xiang Khouang) (Figure 4). It is confined to the montane evergreen forest on limestone mountains at elevations between 850 and 1200 (-1750) m. In part of its distribution along the border between China and Vietnam, it is sympatric with *A. yunnanensis*.

**Table 1.** Morphological character comparison amongst the three regional *Amentotaxus* species.

Characters	*A. hekouensis*	*A. argotaenia*	*A. yunnanensis*
Length of leaf	8–12.5 cm	2–11 cm	3.5–10 cm
Width of leaf	9–14 mm	5–11 mm	8–12 mm
Texture of leaves	thin, leathery	thick, leathery	thick, leathery
Apex of the leaves	long acuminate	rounded to sharply triangular	obtuse or tapered
Width of stomatal bands	2.1–3.0 mm	1–2 mm	3–4 mm
No. rows of each stomatal band	25–30	15–25	ca. 40
Ratio of stomatal band/marginal band	0.75–1	< 1	> 2
Colour of marginal band	bright green in fresh	Yellowish-green	Yellowish-green in fresh
No. raceme per male cone	1–2	2–4 (10)	4–6

#### Phenology.

Male cones of *Amentotaxus hekouensis* mature in March to April; seeds have not been seen so far.

#### Etymology.

The specific epithet refers to the type location in Hekou County.

#### Conservation status.

Several surveys have been carried out in this region since 2006 and only a few small trees of the new species are known. *Amentotaxus hekouensis* occurs in small and isolated patches of forests at very low densities. All the known localities are subject to degradation from surrounding agriculture and forest clearance. Given its montane habitat, the new species may also be susceptible to the impacts of climate change. The combination of a restricted, fragmented distribution with very limited Area of Occupancy, small population size and habitat conversion and destruction throughout its range indicate that its IUCN status should be Endangered under Criterion B2ab(ii,iii) (IUCN 2016b).

## Discussion

*Amentotaxus hekouensis* has distinct morphological features compared to the closely related *A. argotaenia* and the sympatric *A. yunnanensis* (Table 1). Its distinctive nature has also been supported by DNA barcoding analysis of this genus (Figure 1). Therefore, the description of a new species is supported by both morphological and molecular evidence. Due to its restricted distribution, small population size and the prevalence of habitat destruction within its range, *A. hekouensis* is assessed as endangered (EN).

## Supplementary Material

XML Treatment for Amentotaxus hekouensis
